# Exposure of Syrian refugee agricultural workers to pesticides in Lebanon: a socio-economic and political lens

**DOI:** 10.3389/fpubh.2024.1402511

**Published:** 2024-06-27

**Authors:** Bandar Noory, Rima R. Habib, Iman Nuwayhid

**Affiliations:** ^1^Department of Epidemiology and Population Health, Faculty of Health Sciences, American University of Beirut, Beirut, Lebanon; ^2^Department of Environmental Health, Faculty of Health Sciences, American University of Beirut, Beirut, Lebanon

**Keywords:** Syria, refugees, agricultural workers, socio-economic, political, vulnerability, exposure, pesticides

## Abstract

This article adopts a socio-economic and political lens to elucidate the interplay of factors that heighten the vulnerability of Syrian refugee agricultural workers and their exposure to pesticides in Lebanon. It provides a comprehensive understanding for the interconnected social, political and economic factors at the global, regional, national and local levels and how they increase the vulnerability of Syrian refugee agricultural workers, particularly their exposure to pesticides. The global factors highlight the shifts from colonialism to state-controlled economies to neoliberal policies. These changes have prioritized the interests of large agricultural schemes and multinationals at the expense of small and medium-sized agriculture. Consequently, there has been a boost in pesticides demand, coupled with weak regulations and less investment in agriculture in the countries of the Global South. The article explains how the dynamic interaction of climate change and conflicts in the Middle East and North Africa region has negatively impacted the agriculture sector and food production, which led to an increased potential for pesticide use. At the national and local levels, Lebanon’s social, political and economic policies have resulted in the weakening of the agricultural sector, the overuse of pesticides, and the intensification of the Syrian refugee agricultural workers’ vulnerability and exposure to pesticides. The article recommends that researchers, policymakers, and practitioners adopt a political-economic-social lens to analyze and address the full dynamic situation facing migrant and refugee workers in Lebanon and other countries and promote equity in the agricultural sector globally.

## 1 Introduction

Syrian migrant workers have always been a substantial part of the labor force in Lebanon. Following the war in Syria that started in 2011, the number of Syrians who took refuge in Lebanon surged to 1.5 million, constituting close to 30% of its population (1). Of these, less than 800,000 are registered with the United Nations High Commissioner for Refugees (UNHCR) and about 300,000 reside in the Bekaa-Baalbek-Hermel region, the largest agricultural region in Lebanon close to Syria (1), mostly in informal tented settlements and impoverished settings (2). Due to work restrictions and low-cost labor, 24% of the Syrian refugees, including children, work in agriculture (3), which is a known hazardous economic sector, especially for migrants who are exposed to exploitative work conditions including a high risk of exposure to pesticides (3).

Syrian refugee agricultural workers in Lebanon live in suboptimal conditions (3) and are exposed to hazardous working environments which lack basic occupational health and safety (OHS) measures, and where pesticides are excessively used (4, 5). These compounded exposures and vulnerabilities have multiple and concurrent health impacts (4, 6), which have been documented among migrant and refugee workers in other countries (7–11).

This paper suggests employing a socioeconomic and political lens to address an understudied research theme: the socioeconomic and political dynamics that impact the exposure of Syrian refugee agricultural workers to pesticides.

While recent research has expanded its scope to investigate pesticide exposure among agricultural workers, there remains a significant knowledge gap regarding the interconnected political, economic, and social factors that shape the experiences of refugee and migrant workers, including Syrian agricultural workers in Lebanon. Addressing the exposure of Syrian refugee agricultural workers to pesticides is particularly interesting since this population lives and works in a context where multiple instability factors prevail. To the best of our knowledge, no study has explored the impact of socioeconomic and political dynamics on pesticide exposure in the region, and only a few international studies have addressed the wider socioeconomic and political dynamics beyond the workplace.

**Figure 1:**
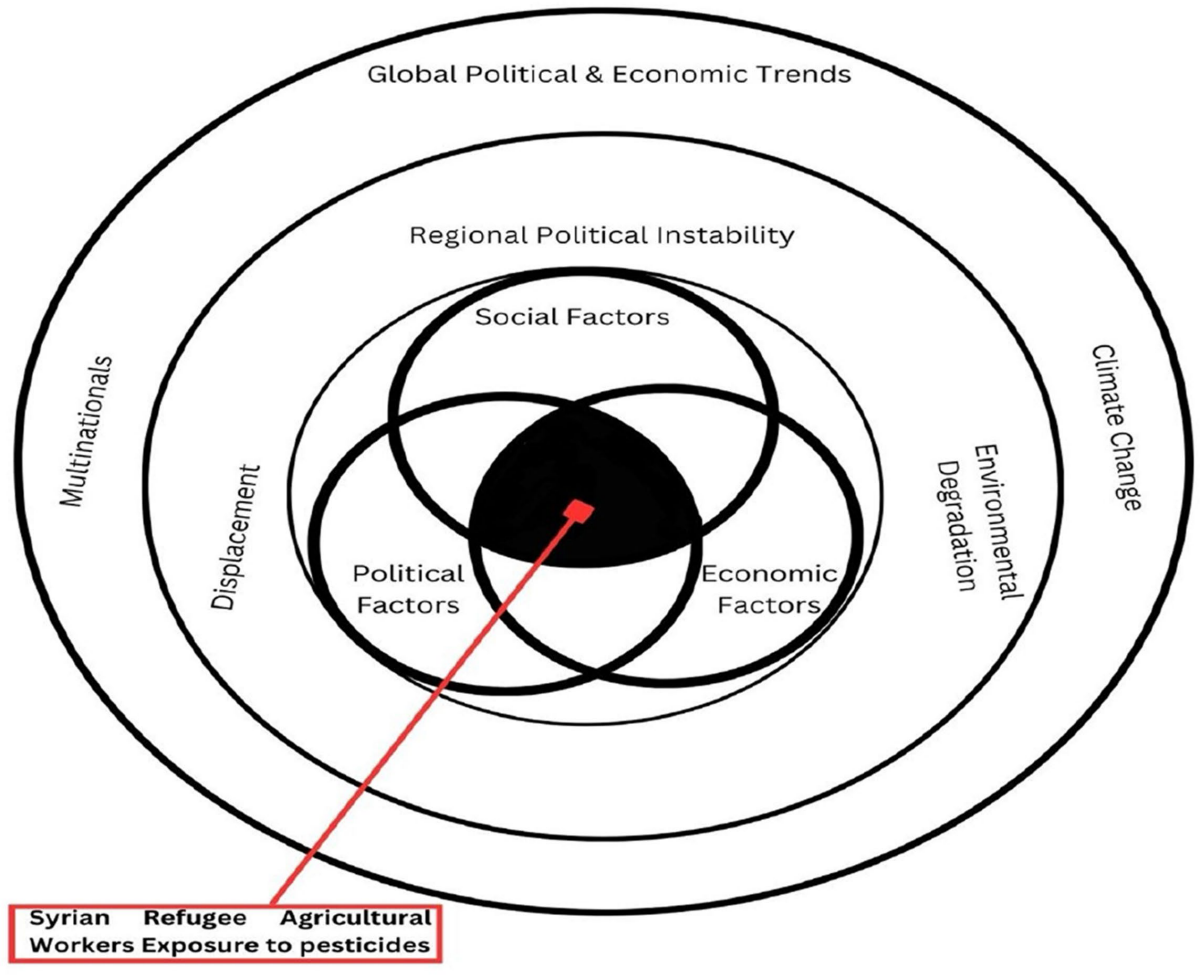
An illustration of political-economic-social factors interaction.

We argue that dynamics at the workplace and exposure to pesticides are determined by distal factors which should be understood to improve the probability of implementing successful health and safety interventions at different levels (12, 13). To make the case, we describe the global economic and political trends and increased use of pesticides, the regional context, and how Lebanon’s political and economic choices have weakened the agricultural sector over time. We then elaborate on how political, economic, and social factors colluded to increase the vulnerability of Syrian refugee agricultural workers and their exposure to pesticides. The diagram in Figure 1 illustrates the interaction between these factors.

## 2 Global trends

Low- and middle-income countries (LMIC) of the Global South have generally experienced challenging political and economic development since World War I (WWI). These countries passed through colonial periods that exploited their natural resources. Following the colonial phase, independence was generally characterized by centralized state-controlled economies that could not withstand their citizens’ internal demands and the external pressure of globalization.

During the 1960s and 1970s, capital-intensive agricultural schemes, promoted through the Green Revolution, replaced traditional farming methods in the Global South, leading to the widespread adoption of chemical-intensive practices (14, 15). This initiative revitalized the pesticide trade, previously in decline due to environmental concerns in the Global North, by creating demand for pesticide use which resulted in a substantial increase in Global South pesticide imports from $641 million a year to $1 billion between 1974 and 1978 (14).

Additionally, the current adaptation to neoliberal economic policies which imposed structural adjustments has led to the reduction in government spending and its control of the economy, the elimination of subsidies, and the adoption of free trade principles (16–19).

Small- and medium-sized agriculture suffered globally at the expense of other economic sectors to the advantage of big agriculture and the multinationals, whose interests were best served in boosting pesticide demand and intensifying agricultural production for cost-effective raw material exports (20–22). Weak regulations, the absence of state subsidies, and cuts to agricultural budgets dominated the scene in countries of the Global South. Together with unequal global trade relationships with high-income countries (HIC), which continued to subsidize their agricultural sectors (23), this weakened their competition in global market and reinforced their dependency. This trend was witnessed in Brazil and Ecuador where a significant portion of export earnings was allocated to pesticide imports (24, 25). Furthermore, HIC monopolized technologies through intellectual property rights and consequently crop production; the productivity ratio between HIC and LMIC increased from 10:1 in 1940 to 2000:1 in 2012 (23, 26). This emerging global order has exposed the most vulnerable people in the social hierarchy (27), especially agricultural workers who are directly exposed to pesticides and other occupational hazards, especially that the capitalists of LMIC further exploit agricultural workers to decrease production costs and remain competitive in the global market (28).

Furthermore, migrant agricultural workers are exposed to an expanding and competing capitalist system, often at the expense of certain ethnic or cultural groups whose low wages are justified through biological determinism (14). Following the 2008 financial crisis, Europe witnessed a rise in far-right political ideologies and wider discrimination and racism against migrants portrayed as culturally, religiously, and nationally different (29). These ideologies introduced fear from the “other” by depicting migrants as a threat to jobs and social security systems, emphasizing cultural differences to justify exclusion (30). Ironically, even some environmental-friendly initiatives have happened at the expense of migrant workers, such as in the banning of environmentally persistent organochlorine pesticides which were replaced with the less persistent but more acutely toxic organophosphates to which migrant workers are exposed (14). Similarly, the COVID-19 pandemic has affected labor dynamics disproportionally, with the already marginalized migrant agricultural workers being impacted more severely than other workers (31). The inadequacy of labor regulations to protect migrant and seasonal workers became evident in these settings, further highlighting the vulnerability of these populations (31).

Sadly, climate change, also linked to aggressive and greedy global economic policies, is undermining food security and feeding into the cycle of overuse of pesticides to meet increasing food needs (32).

## 3 Regional context

The Arab region, to which Lebanon belongs, is heterogeneous politically and economically (33). It includes some of the wealthiest countries globally as well as some of its poorest. Huge inequalities have been documented between and within these countries. The region is the least endowed in natural sources of water globally putting it at an increased risk of food insecurity (34). Furthermore, the region is witnessing wide environmental degradation and is threatened by climate change, reflected in increased number of heat waves, drought, and sandstorms (35). The situation is further complicated by political instability and multiple concurrent wars and civil conflicts that hinder sustainable development (36). Forced migration due to climate change and conflict is the highest in the world. Almost more than 11 million internally displaced people reside in the Middle East and North Africa (MENA) region (37). These dynamic and interconnected factors have a negative impact on agriculture in the region and consequently on food production and the potential to use more pesticides.

## 4 Lebanon: the case of Syrian refugee agricultural workers

Lebanon shares many of the experiences mentioned before but its economic and political development during and after the French colonial period (1917–1943) has been different from other countries in the region due to its unique history, geography, and community composition. The following sections will examine these developments in Lebanon and how together with social factors they have impacted the agriculture sector and the use of pesticides and intensified the vulnerability of Syrian refugee agricultural workers.

Lebanon was declared an independent state separate from Syria under the French Mandate in 1920, a few years after the end of WWI. This country of 10,452 km^2^ lies at the eastern shores of the Mediterranean and shares borders with Syria in the north and east, and Palestine in the south. Since its declaration, Lebanon became a parliamentary democratic republic, however based on a confessional political system where high-ranking public offices are distributed between the different Christian and Muslim sects (38). The country’s sensitivity to demographic changes is rooted in its sectarian balance. This has influenced debates over the country’s identity and its relationships with the Arab world, the West, and the East. Additionally, it has fueled resistance to nationalize non-Lebanese, regardless of their residency duration. This policy has been applied strictly to Palestinian refugees who entered Lebanon since 1948 and Syrian refugees since 2011, and it also restricted their work permits to construction, agriculture, and garbage collection.

Lebanon suffered from a 15-year civil war (1975–1990) and post-war political polarizations, fueled by the internal sectarian divisions and the ongoing Israeli-Palestinian/Arab conflict that resulted in recurrent Israeli invasions (1969, 1978, 1982, 1996, 2006), a long-term Israeli occupation of south Lebanon (1978–2000), and a 30-year Syrian political and military control of the state (1976–2005). Lebanon, particularly the Bekaa and Akkar regions, attracted many Syrian refugees who fled the war since 2011.

This brief is meant to contextualize some of the political, economic, and social factors behind the Syrian refugee vulnerabilities, divided into (1) *structural vulnerabilities* that include the national political and economic factors and choices that have impacted the agriculture sector and resulted in the absence of OHS regulations and the increased use of pesticides, and (2) *social vulnerabilities* that result from the structural ones but manifest at the local level and impact the Syrian refugee agricultural worker directly.

### 4.1 Structural vulnerabilities (political and economic factors)

Lebanon’s free market policies have led to weakened state institutions, a focus on services, and a decline in agriculture (39–41). The subsequent state’s neglect of agriculture has benefited elites and multinational companies, including those in the agricultural and pesticide sectors (40).

Historically, the imposition of a dependency model through the export of agricultural raw materials, mainly silk, has occurred between 1860 and 1914, and served the interests of French industrial capital accumulation (40, 42). During this period, crops at a large-scale shifted from subsistence to cash crop agriculture (40). In 1895, due to a decline in silk production profitability, the French decided to transition to more lucrative crops such as grapes, citrus, olives, and tobacco (40). Other examples of dependency occurred after independence in 1943. Between 1946 and 1980, Lebanon received $86.2 million in food aid leading to the “wheatification” of its diet (40). Notably, high-yielding crop cultivation for export, such as fruits, apples, and citrus, increased by 700% in 1971 in contrast to 250% from 1951 to 1971 (40). Agricultural restructuring efforts, responsive to global demand, targeted more profitable crops, thereby boosting the need for pesticides to improve productivity. Pesticides and agrochemicals became a profitable market controlled by foreign companies, resulting in a 300% increase in pesticide sales in Lebanon by 1975 (40).

The agriculture sector’s contribution to the Gross Domestic Product (GDP) declined significantly with time. In 2016, the service and banking systems contributed 74.2% to GDP, which increased to 94% in 2021, while that of agriculture dropped from 2.7 to 1.4% during the same period (40). The agricultural sector’s decline is evident in the government’s decrease in spending, from 2.3% in 1973 to 1.4% in 2021, and the drop in active population engaged in agriculture, from 48.9% in 1959 to 18.9% in 1970 (40). Furthermore, the fragile state nurtured a hybrid political and economic structure led by a sectarian oligarchy in a mix of concurrent feudal, modern, and neoliberal policies (43). This translated into an extreme divide between the stronger urban centers and the marginalized rural peripheries (44). Inequality was a main feature that resulted in recurrent political instabilities, urban–rural migration, emigration, and demographic changes that continue to shape Lebanon.

The recent withdrawal of agriculture can be traced back to the 1992 reform, which shifted financing from cash to credit based. Without a state-supported credit system, high-interest loans from merchants left farmers, especially small farmers, unable to repay due to rising costs and decreased agricultural yield. As a result, farmers’ debts rose to $60 million in 2019 (40). This was further exacerbated by the neo-liberalization project, which was financed through public debt, reaching 120% of the GDP in 1993, and then topping 171% of the GDP in 2020 (39, 40).

The Lebanese state has historically favored large-scale farmers by granting them more loans, hence disadvantaging small farmers (45). There is evidence that increased debt relations between farmers, agrochemical and agribusiness companies, and brokers have led to pesticide overuse in Lebanon (46). While pesticides may boost yields and lower production costs in the short term, over time they can lead to decreased yields and increased costs of production, further deepening indebtedness and leading to more pesticide use (47). Furthermore, free trade policies and economic liberalization led to an increase in agricultural production cost (fuel, pesticides, fertilizer) (48). Hence, small-scale farmers faced debt, forcing them to sell their lands, move to urban areas, and experience unemployment and poverty (49). This led to a concentration of wealth among a minority. In 1970, 20–25 traders dominated the citrus and apple markets, controlling 80 and 67% of respective industries. This allowed them to purchase commodities cheaply from farmers and make significant profits (40). At the same time, land ownership became increasingly concentrated in the hands of a few individuals and 50% of local population owns only one hectare (40).

The capital-intensive agricultural system in Lebanon has substantially relied on pesticide use which has increased from 4 kg/hectare of cropland in 1990 to 6.5 kg/hectare in 2021, nearly three times the global average of 2.26 kg/hectare in 2021 (50). Agricultural workers hence faced increased exposure to harmful pesticides, exacerbated by weak or non-existent OHS regulations.

In Lebanon, OHS has been marginalized as a result of neoliberal trends. Important OHS-related International Labor Organization (ILO) conventions have not been ratified by the Lebanese State. For example, Lebanon failed to ratify two ILO Conventions pertaining to OSH in the agriculture sector: C184 (2001) on Safety and Health in Agriculture and C129 (1969) on Labor Inspection (Agriculture) (5). The ratification of these conventions is followed by the incorporation of its provisions into updated national legislations. Signing these conventions is thus an essential stage in safeguarding agricultural workers, especially that the Lebanese Labor Law does not cover the agricultural sector, which puts agricultural workers at greater risk of exploitation and exposure to hazards (39). Furthermore, new imported agricultural technologies and chemicals marketed by multinational corporations and local partners, have shifted farmers, in Lebanon and globally, away from traditional, environmental-friendly practices toward practices that promote pesticide reliance and overuse (20, 48, 51, 52). Concurrently, large landowners and agricultural elite have opposed policies regulating pesticide use (46), putting the marginalized Syrian refugee agricultural workers at greater risk of exposure to pesticides without adequate preventive measures in place.

### 4.2 Social vulnerabilities

Lebanon’s political and economic policies have led to the marginalization of Syrian refugees whose increased numbers provided a surplus in labor force, for a struggling agricultural sector. Since Syrian refugees have minimal basic rights and their work is restricted by law to a few occupations including agriculture, and since their majority lack work permits, they have no option but to work in agriculture under precarious conditions and at low wages. They live in a cycle of poverty and exploitation, further manifested by an increase in child labor among Syrian children (53). A study on 4,090 school-aged Syrian refugee children revealed that 82% do not attend school and 76% seek unskilled labor opportunities in agriculture (2). Furthermore, Syrian agricultural workers and their families live in informal tented settlements which are managed from within by a person, called *Shaweesh*, with good social capital and connections (54). The *Shaweesh*, usually a man, acts as the refugees’ work agent, where he is contacted by farm owners to request laborers as needed. The *Shaweesh* identifies and transports the refugee workers from the camp, supervises them, and takes a cut from their wages (up to 30%), contributing to their exploitative working conditions. Syrian agricultural workers work longer hours and are paid less than their Lebanese counterparts. Among the Syrian workers, females receive 30–50% of the daily wage received by males, and males working in large-scale farms may be paid twice those working in small-scale farms (3). In some cases, workers are paid on piecework basis, which could lead to work intensification (2), and an increased exposure to pesticides.

In addition, as mentioned before, agricultural workers are not covered by OHS laws. They also lack legal and political agency compounded by poverty, poor education, and limited access to resources. These factors negatively impact their autonomy, decision-making, risk perception, attitudes toward pesticides, OHS knowledge, proper use of personal protective equipment (PPE), and standardized pesticide use (5). If not provided by the farmers, workers cannot afford acquiring PPE (55). In fact, Syrian refugee agricultural workers who use pesticides rarely receive OHS training. Only a third uses masks, many use their bare arms to mix pesticides, and all take their work clothes home for washing, thus exposing family members to harmful exposures (5). Even empty pesticide containers are dumped in the fields or are collected by workers or their children for reuse or sale (5).

Agricultural workers also have no access to social protection and comprehensive health coverage and they have to pay 20–25% out-of-pocket for health services provided by the UNHCR (56, 57). This adds to their stressors and curtails their right to proper medical management and work compensation in case of work-related health problems, including those related to pesticide exposure (3, 58). These deprivations increase Syrian refugee workers’ vulnerability and their likelihood of exposure to pesticides and missing timely health interventions.

Numerous studies across Europe, the United States, and Canada have explored the economic vulnerability of migrant agricultural workers, who often endure precarious working conditions, including long hours, low pay, and temporary employment arrangements (11, 59). Temporary workers, such as seasonal and daily agricultural workers, are particularly at risk, facing higher pesticide exposure and hesitating to request safety equipment due to job insecurity (60–62). Additionally, migrant agricultural workers often engage in piecework for long hours and involve their children in agricultural activities to supplement family income increasing their vulnerability to pesticides (63). The interplay between economic and political vulnerabilities is evident in all these studies. Showing that migrant workers without legal status receive below-minimum wages and are more exposed to pesticides (64, 65).

## 5 Conclusion and implications

This paper illustrates that agricultural OHS and exposure to pesticides are not technical problems that could be resolved at the local farm level. Although OHS training, the provision of basic OHS protection, and the adoption of engineering solutions are helpful and may reduce the risk of workers’ exposure to pesticides and other agricultural hazards, impactful and sustainable OHS programs demand interventions at the root causes, i.e., the political, economic, and social factors. To date, Lebanon’s political and economic policies had undermined good governance, marginalized the agriculture sector, and constructed work and social environments for the refugees that increased their vulnerabilities. These policies are not unique to Lebanon but mirror the main features of global capitalism and neo-liberalism where intensive food production that is controlled by the few requires intensive pesticide use. It is reported that large-scale agricultural schemes have increased pesticide use by 1.8% for every 1% increase in crop output in high, middle, and low-income countries (66). Scholars in Lebanon and global safety initiatives have repeatedly recommended the need to adopt new policies, remove banned pesticides, and switch to safer alternatives (26, 52). However, the situation continues to deteriorate and the vulnerability of Syrian refugees to pesticides and other hazards keeps increasing. Deregulation and reduced state involvement have to be reversed since they lead to unsafe agricultural practices and worse exploitation of Syrian workers, but this would necessitate a holistic social-centered approach.

Minimizing the use of pesticides at the farm and workers’ levels also has implications for the general population. Overuse of pesticides pollutes the ecosystem (soil and water resources) and reaches the consumer as pesticide residues or contaminated water sources (67, 68). Hence, efforts to minimize pesticide use would require a different mindset and a new school of thought at the local, regional, and global levels. Sadly, the reverse is still happening, such as in Brazil where lobbying led to the design of agrarian policies in favor of large-scale farming and pesticide application (24).

This paper has focused on the case of Syrian refugee agricultural workers in Lebanon but its findings carry wider and international implications. Future research on refugee workers’ health should address the root causes of exposure to pesticides by considering the broader structural dynamics on a global scale and adopting a political-economic-social lens to pesticide use.

## Data availability statement

The original contributions presented in the study are included in the article/supplementary materials, further inquiries can be directed to the corresponding author.

## Author contributions

BN: Conceptualization, Methodology, Visualization, Writing – original draft, Writing – review & editing. RH: Conceptualization, Funding acquisition, Methodology, Supervision, Visualization, Writing – review & editing. IN: Conceptualization, Funding acquisition, Methodology, Supervision, Visualization, Writing – original draft, Writing – review & editing.
